# Lean and/or Six Sigma for process optimization in the perioperative period: an integrative review

**DOI:** 10.1590/0034-7167-2023-0431

**Published:** 2024-06-14

**Authors:** Lucas Gardim, Fernanda Rodrigues dos Santos, Bruna Moreno Dias, Lívia Barrionuevo El Hetti Fuentes, Renata Cristina de Campos Pereira Silveira, Andrea Bernardes

**Affiliations:** I Universidade de São Paulo. Ribeirão Preto, São Paulo, Brazil

**Keywords:** Surgicenters, Total Quality Management, Process Optimization, Perioperative Period, Health, Centros Quirúrgicos, Gestión de la Calidad Total, Optimización de Procesos, Periodo Perioperatorio, Salud

## Abstract

**Objective:**

To analyze the evidence on the influence of Lean and/or Six Sigma for process optimization in the perioperative period.

**Methods:**

Integrative review carried out in the MEDLINE (PubMed), Web of Science, EMBASE, CINAHL, Scopus and LILACS databases on the use of Lean and/or Six Sigma to optimize perioperative processes. The studies included were analyzed in three thematic categories: flow of surgical patients, work process and length of stay.

**Results:**

The final sample consisted of ten studies, which covered all operative periods. Lean and/or Six Sigma make a significant contribution to optimizing perioperative processes.

**Final considerations:**

Lean and/or Six Sigma optimize perioperative processes to maximize the achievement of system stability indicators, making it possible to identify potential problems in order to recognize them and propose solutions that can enable the institution of patient-centered care.

## RESUMEN

**Objetivo:**

Analizar las evidencias sobre la influencia del Lean y/o Six Sigma para optimizar los procesos en el periodo perioperatorio.

**Métodos:**

Es una revisión integradora llevada a cabo en las bases de datos MEDLINE/PubMed, Web of Science, EMBASE, CINAHL, Scopus y LILACS, sobre la utilización del Lean y/o Six Sigma para optimizar los procesos en el perioperatorio. Los estudios incluidos se analizaron en tres categorías temáticas: flujo de pacientes quirúrgicos, proceso de trabajo y tiempo de la estancia.

**Resultados:**

La muestra final estuvo formada por diez estudios, los cuales contemplaron todos los períodos operatorios. Lean y/o Six Sigma contribuyen grandemente para mejorar los procesos en el perioperatorio.

**Consideraciones finales:**

Lean y/o Six Sigma optimizan los procesos perioperatorios al maximizar el alcance de los indicadores de estabilidad de los sistemas, facilitando la identificación de problemas potenciales para reconocer y proponer soluciones que ayuden a instituir un cuidado más centrado en el paciente.

## INTRODUCTION

Scientific evidence demonstrates the effectiveness of Lean and/or Six Sigma when applied to the healthcare scenario^(1, 2)^. In the perioperative context, work and patient processes are highly complex^(3)^, due to the fact that it encompasses several professionals and interacts with different sectors within the hospital. For this reason, it is imperative to optimize processes relating to the flow of work and patients, especially with the aim of reducing length of stay and achieving positive health outcomes.

In the mid-1980s, with its origins in the Toyota Production System, Lean was established as a philosophy that focuses on reducing waste (waiting time, unnecessary human movement, unnecessary transportation, rework, ultra-processing, overproduction and stock) and establishing processes that add value^(4)^. From another perspective, originating at Motorola, Six Sigma is a methodology that focuses on reducing variability in processes. Given their complementarity, the Lean philosophy and the Six Sigma methodology have been brought together in “Lean Six Sigma”^(5)^.

When applied to patient optimization and discharge processes, intensive care units, emergency departments, operating rooms and other units, Lean and/or Six Sigma make it possible to reduce length of stay^(6)^, contributing to improving patient safety, minimizing perceived discomfort and even reducing costs for the healthcare organization^(7, 8, 9, 10, 11, 12, 13, 14)^.

In addition, when it comes to the perioperative period, studies have indicated the influence of implementing Lean and/or Six Sigma in reducing operating room turnaround times, guaranteeing the provision of an efficient service^(15, 16, 17)^. Therefore, in order to propose effective management, it is essential to identify procedural limitations that can enable processes optimization, i.e. reduce time and procedural variability, improving the efficiency of the operating room^(18, 19)^.

Even though surgical care consumes a large amount of technological resources and is a huge burden for the health organization^(20)^, not all of the costs incurred in performing surgical procedures are in fact necessary, given that they are directly related to the way in which operating rooms are managed. However, the adoption of Lean and/or Six Sigma has the potential to make it possible to systematize processes and reduce costs for the hospital organization, not only transforming the quality of care, but also increasing patient and professional satisfaction rates^(19, 21, 22, 23)^.

In this sense, given the potential of Lean and/or Six Sigma to achieve excellent quality indicators in the perioperative context^(24, 25)^, it is essential to synthesize the scientific evidence that proves such effectiveness, in order to endorse the implementation of such methodologies in different hospital organizations, especially with regard to surgical care, with an influence on the health outcomes achieved by patients.

## OBJECTIVE

To analyze the evidence on the influence of Lean and/or Six Sigma for optimizing processes in the perioperative period.

## METHODS

Integrative review following the methodological framework of Whittemore and Knafl (2005)^(26)^ and reported according to Preferred Repor ting I tems for Systematic Review and Meta-Analysis (PRISMA)^(27)^.

The guiding question was outlined according to the acronym PICo (P= Population; I= Interest; Co= Context)^(28)^. Thus, “P” was assigned to process optimization; “I” to Lean and/or Six Sigma; and “Co” to the perioperative period, including pre-, intra- and post-operative. Thus, the following guiding question was established: “What is the influence of Lean and/or Six Sigma on process optimization in the perioperative period?”

The search was performed in August 2021 and updated in April 2023 in the following databases: Medical Literature Analysis and Retrieval System Online (MEDLINE/PubMed), Web of Science, EMBASE, Cumulative Index to Nursing and Allied Health Literature (CINAHL), Scopus and Latin American and Caribbean Health Sciences Literature (LILACS).

The controlled and non-controlled descriptors were established in accordance with the Medical Subject Headings (MeSH), Emtree, CINAHL Subject Headings and Health Sciences Descriptors (DeCS). In order to guarantee the sensitivity of the search strategy in the selected databases, this process was carried out with the support of a librarian with expertise in bibliographic searching. Chart 1 shows the search strategies applied to each database.

**Chart 1:** Search strategy in the selected databases

Database	Search strategy
MEDLINE (PubMed)	(“Total Quality Management” OR “Lean Healthcare” OR “Lean System” OR “Lean methodology” OR “Lean Six Sigma” OR “Six Sigmas” OR “Six Sigma”) AND (Surgicenter* OR “Surgery Department”)
*Web of Science*	(“Total Quality Management” OR “Lean Healthcare” OR “Lean System” OR “Lean methodology” OR “Lean Six Sigma” OR “Six Sigmas” OR “Six Sigma”) AND (Surgicenter* OR “Surgery Department”)
EMBASE	(“Total Quality Management” OR “Lean Healthcare” OR “Lean System” OR “Lean methodology” OR “Lean Six Sigma” OR “Six Sigmas” OR “Six Sigma”) AND (Surgicenter* OR “Surgery Department”)
CINAHL	(“Total Quality Management” OR “Lean Healthcare” OR “Lean System” OR “Lean methodology” OR “Lean Six Sigma” OR “Six Sigmas” OR “Six Sigma”) AND (Surgicenter* OR “Surgery Department”)
SCOPUS	(“Total Quality Management” OR “Lean Healthcare” OR “Lean System” OR “Lean methodology” OR “Lean Six Sigma” OR “Six Sigmas” OR “Six Sigma”) AND (Surgicenter* OR “Surgery Department”)
LILACS	(“Total Quality Management” OR “Lean Healthcare” OR “Lean System” OR “Lean methodology” OR “Lean Six Sigma” OR “Six Sigmas” OR “Six Sigma”) AND (Surgicenter* OR “Surgery Department”)

Regarding the inclusion criteria, were included: original articles, published between 2010 and 2023 and without language restrictions, which evaluated the influence of Lean and/or Six Sigma for process optimization in the perioperative period. Literature reviews and grey literature were excluded.

The time frame was decided based on the timeline for implementing Lean and/or Six Sigma in the healthcare sector. In fact, there is no consensus in the literature regarding the exact year in which the implementation process began; however, it is estimated that it took place around the 2000s. As in the United States of America, the first event to disseminate Lean and/or Six Sigma took place in 2010^(29)^, while in Brazil, it is estimated that the process started in 2013^(30)^, denoting inequality not only in the implementation, but also in the dissemination of the topic. For this reason, in the case of a change management process, with regard to the culture of healthcare organizations, for the implementation of Lean and/or Six Sigma, it was estimated that the indicators relating to the implementation could be denoted around 2010, especially in the perioperative context.

The studies identified in the databases were exported to Rayyan QCRI^(31)^ for the removal of duplicate studies and screening. Three stages of selection were then carried out independently by two reviewers.

In the first stage, after removing duplicates, the titles and abstracts of the identified studies were assessed. They were then read in full to confirm their eligibility. Disagreements between the reviewers were resolved through peer meetings. On the occasions when the disagreements were not resolved, a third reviewer with expertise in the subject was required. Finally, data was extracted independently from the studies selected for the sample, taking into account: authorship, year of publication and country in which the study was conducted; methodology used (Lean Healthcare, Six Sigma or Lean Six Sigma) and operative period (preoperative, intraoperative and/or postoperative); tools used and population; results achieved and outcome.

For the final sample, the strength of evidence was analyzed, as proposed by Melnyk & Fineout-Overholt (2011)^(32)^. The studies were evaluated according to three hierarchies: (1) Intervention or Diagnosis/Diagnostic Test, with seven levels; (2) Prognosis/ Prediction or Etiology, with five levels; and (3) Significance, with five levels. For the three hierarchies, the lower the level, the stronger the evidence^(32)^.

In addition, the Critical Appraisal Checklist for Studies Reporting Prevalence Data, recommended by the JBI, was used to assess methodological quality^(33)^. Therefore, the JBI Critical Appraisal Checklist for Analytical Cross Sectional Studies tool was used for the cross-sectional studies^(34)^; for cohort studies, the JBI Critical Appraisal Checklist for Cohort Studies tool was used^(34)^; for qualitative studies, the JBI Critical Appraisal Checklist for Qualitative Research tool was used^(35)^; for the quasi-experimental studies, the JBI Critical Appraisal Checklist for Quasi-Experimental Appraisal Tool was used^(36)^. In the methodological evaluation of the studies, it is clear that a higher number of answers classified as “Yes” indicates better methodological quality, according to the tools proposed by the JBI^(33)^.

It should be noted that the processes of assessing the strength of evidence and methodological quality were carried out independently by two reviewers. Disagreements with the preliminary results were resolved by a third reviewer with methodological expertise. Then, the data was analyzed using descriptive and content analysis, which included frequency counts and descriptions on the flow of surgical patient, work process and length of stay.

## RESULTS

**Figure 1:**
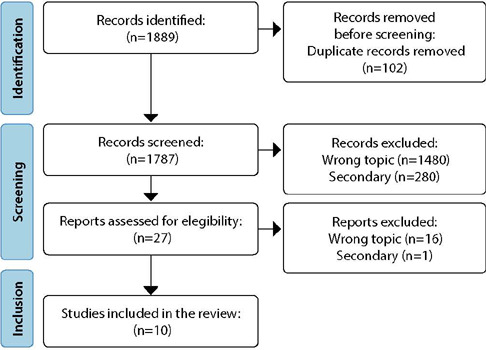
Flowchart for selecting studies according to the PRISMA flowchart^(27)^

A total of 1889 records were identified in six databases. Following the article selection flowchart, ten articles were included in this review^(9, 37, 38, 39, 40, 41, 42, 43, 44, 45)^, according to Figure 1.

In the final sample, four (40%) were developed on the American continent (one in Brazil^(39)^, one in Canada^(43)^, one in the United States of America^(37)^ and one in Peru^(40)^), four (40%) were developed on the European continent (one from Ireland^(38)^, one from Italy^(9)^, one from United Kingdom^(45)^ one from Switzerland^(41)^) and two (20%) from Asia (one in Saudi Arabia^(44)^ and one in Taiwan^(42)^). Regarding the years of publication, there was a predominance of studies published in 2015 (n=2), corresponding to 20% of the sample.

Of the final sample, five studies (50%) adopted Lean Healthcare^(38, 39, 41, 43, 45)^ to evaluate process optimization, three (30%) applied Six Sigma^(37, 42, 44)^ and two (20%) Lean Six Sigma^(9, 40)^. Thus, it was observed that these methodologies have a diversity of application in the operative periods, since all of them were contemplated, namely: preoperative (n=5)^(9, 37, 38, 39, 40)^, intraoperative (n=6)^(9, 37, 38, 40, 41, 42)^ and post-operative (n=6)^(9, 37, 38, 43, 44, 45)^. It should be emphasized that the sum of the studies exceeds the number included in the final sample, considering that three studies cover the perioperative period^(9, 37, 38)^ and one covers the pre- and intra-operative periods^(40)^. Chart 2 summarizes the manuscripts analyzed.

**Chart 2:** Summary of the articles included in the integrative literature review

Operative period	Author / Country	Methodology	Intervention	Results	Outcome: Have it optimized the processes?	Level of Evidence
**Perioperative**	Amato-Vealey et al., 2012^(37)^ United States	Six Sigma	Tools: patient flow mapping, brainstorming, multidisciplinary visit. Population: not specified.	The implementation of Six Sigma resulted in improvement in the discharge process and ensured the ability to maintain a continuous flow of surgical patients without incurring financial costs.	Yes	VI*
	Montella et al., 2017^(9)^ Italy	Lean Six Sigma	Tools: Define-Measure-Analyze-Improve-Control (DMAIC), process mapping, brainstorming and fishbone. Population: 20,000 patients.	The actions resulted in a significant reduction in the average number of hospitalization days (from 45 to 36 days). There was also a reduction in the percentage of patients colonized by sentinel bacteria (from 0.37% to 0.21%).	Yes	II*
	Ullah et al., 2020^(38)^ Ireland	Lean Healthcare	Tools: 5s, Kaizen, pull and push systems and process mapping. Population: Phase 1: 100 patients and 20 employees (clerical, administrative and staff ) interviewed in order to understand their perspectives on the areas that required attention to improve service delivery and efficiency; Phase 2: 100 patients.	The results show the patient’s “perception” of waiting time. In phase 2, perception improved with 83% of patients compared to 67% in phase 1; perception of waiting time for medical review also improved significantly, with 26% of patients reviewed in phase 2 compared to 0% in phase 1. Only 7% of patients had a total stay of 6-8h in phase 2 compared to 33% in phase 1. There was satisfaction in different domains, perceptions of privacy and overall treatment was also improved.	It cannot be said that processes have been optimized.	VI*
**Preoperative**	Godinho Filho et al., 2015^(39)^ Brazil	Lean Healthcare	Tools: 5s e *Single Minute Exchange of Dies* (*SMED*). Population: quality manager (n=1), quality analyst (n=1), coordinator of the Materials and Sterilization Center (CME) (n=1), head nurse of the operating room (n=1), external consultants (unspecified), solution manager (n=1), consultant analysts (n=2).	A significant 94% reduction in the surgical delay rate due to a lack of materials in the Sterilization Materials Center (CME) was observed after the Kaizen event. Finally, a significant reduction in postoperative infection can be seen due to improved flow and standardized operating procedures, dropping from 1 to 1.5% to 0.21%.	Yes	VI*
**Preoperative and Intraoperative**	Iverson et al., 2021^(40)^ Peru	Lean Six Sigma	Tools: Plan-Do-Study-Act (PDSA), process mapping and fishbone. Population: 1- Pre-intervention: 75 patients (53 emergency and 22 elective). 2- Post-intervention: 109 patients (111 emergency and 31 elective).	Patients (87%) on the new waiting list had all relevant clinical data documented, a 13.3% improvement on the pre-existing list. The time from admission to discharge for all surgeries improved from 5 to 4 days (p<0.05) after the intervention.	Yes	II*
**Intraoperatório**	Amati et al., 2022^(41)^ Switzerland	Lean Healthcare	Tools: Gemba walks, process mapping, root cause analysis and Single Minute Exchange of Dies (SMED). Population: multidisciplinary team (a chair of anesthesia, a nurse anesthetist, a surgical technician, a housekeeper, an operating room administrative secretary, two surgeons and two head nurses, representing the gynecology and general surgery specialties).	The changeover time between operations was reduced by 17 minutes for gynecology and 15 minutes for general surgery (25% on average), with no changes in terms of infrastructure, technology or resources.	Yes	VI*
	Leu et al., 2013^(42)^ Taiwan	Six Sigma	Tools: process mapping. Population: Phase 1: 1246 patients. Phase 2: 1266 patients.	The reduction in waiting time for each study group was 0.54, 4.23 and 7.82 minutes, respectively. The average surgery turnover time also decreased significantly for each group (11.98, 16.4 and 19.72 minutes, respectively). The Six Sigma quality indicator changed from 3.35δ to 3.46δ, a difference of 0.11δ.	Yes	II*
**Post-surgery**	Blouin-Delisle et al., 2018^(43)^ Canada	Lean Healthcare	Tools: Define-Measure-Analyze-Improve-Control (DMAIC), Kaizen workshop, process mapping. Population: Project Care Unit professionals: head nurse (n=2), nurses (n=3), clerical assistant (n=1), head of admissions (n=1), head of surgical ward (n=1), agent responsible for implementing Lean concepts (n=1).	Hospital 1: The length of stay in the recovery ward was reduced by 62 minutes (68% reduction) and there was an increase of around 25% of all admissions made during the day after the project compared to the period before the project. Hospital 2: For the HEJ Lean project, time in the recovery ward was reduced by 6 minutes (29% reduction).	Yes	VI*
	Bouras, 2015^(44)^ Saudi Arabia	Six Sigma	Tools: process mapping, fishbone, root cause analysis, cause and effect diagram, brainstorming. Population: not specified.	The results show a reduction in cycle time, impacting the reduction in the time needed to contact doctors; minimizing the time needed to carry out urgent cases and the patient referral process; reducing the delay time before starting the execution of the treatment plan.	Yes	IV**^†^**
	McCulloch et al., 2010^(45)^ United Kingdom	Lean Healthcare	Tools: 5S, process mapping, visual management and Plan-Do-Check-Action (PDCA). Population: 1- Pre-intervention: 607 patients. 2- Post-intervention: 602 patients.	Focus on establishing safe processes and better results. The proportion of patients requiring transfer to other wards fell from 27% to 20%. Length of stay proved to be the most important risk factor for patient safety incidents.	It cannot be said that processes have been optimized.	IV*

*Intervention or Diagnosis / Diagnostic Test; ^†^Prognosis / Prediction or Etiology.

In terms of quality analysis, of the cross-sectional studies (n=4), two received a total of six “yes” answers; of the qualitative studies (n=2), only one received a total of eight “yes” answers; of the quasi-experimental studies (n=3), two received a total of eight “yes” answers; and finally, of the cohort study (n=1), the article included received a total of nine “yes” answers. Chart 3 shows the methodological evaluation broken down by tool and methodological design. It should be noted that none of the included studies received a total of “yes” answers, considering the number of questions included in each instrument.

**Chart 3:** Methodological Evaluation using the JBI Critical Appraisal Checklist for Studies Reporting Prevalence Data.

Cross-sectiona^l(34)^	Q1	Q2	Q3	Q4	Q5	Q6	Q7	Q8	Q9	Q10	Q11	Total (Yes)
Amato-Vealey et al., 2012^(37)^	N*	Y†	Y	Y	N	U‡	Y	U	-	-	-	4
Blouin-Delisle et al., 2018^(43)^	Y	Y	Y	Y	U	U	Y	Y	-	-	-	6
Bouras, 2015^(44)^	N	U	Y	Y	N	N	Y	Y	-	-	-	4
Ullah et al., 2020^(38)^	Y	Y	Y	Y	N	N	Y	Y	-	-	-	6
**Qualitative** ^(36)^												
Amati et al., 2022^(41)^	Y	Y	Y	Y	Y	Y	U	NA^§^	Y	Y	-	8
Godinho Filho et al., 2015^(39)^	Y	Y	Y	Y	Y	Y	U	U	N	Y	-	7
**Quasi-experimental** ^(36)^												
Iverson et al., 2021^(40)^	Y	Y	Y	N	Y	Y	Y	Y	Y	-	-	8
Leu et al., 2013^(42)^	Y	Y	Y	N	Y	Y	Y	Y	Y	-	-	8
Montella et al., 2017^(9)^	Y	Y	Y	N	N	Y	Y	Y	Y	-	-	7
**Cohort** ^(33)^												
McCulloch et al., 2010^(45)^	Y	Y	Y	U	Y	Y	Y	Y	Y	NA	Y	9

*N= No; ^†^Y= Yes; ‡U= Unclear; ^§^NA= Not Aplicable.

## DISCUSSION

In order to analyze the publications included in this study and outline the theoretical construct, the selected articles were organized into three thematic categories, namely: flow of surgical patients^(37, 40, 41, 42, 43)^, work process^(39, 44)^ and length of stay^(9, 38, 45)^.

### Surgical Patient Flow

Considering all the studies included in this review, five analyzed the influence of Lean and/or Six Sigma with regard to optimizing the flow of surgical patients^(37, 40, 41, 42, 43)^.

In the perioperative context, it is essential that the flow of surgical patients is optimized so that waiting times for surgical procedures are minimized, even for general elective procedures, with a view to patient experience and safety. In this sense, considering the principles of the culture of continuous improvement encompassed by Lean, one should always aim to promote lean patient care, guaranteeing the necessary resources at the ideal time, avoiding unnecessary human movement and transportation.

In fact, Six Sigma has the potential to guarantee a continuous flow of surgical patients, reducing the time from admission to discharge for various surgical procedures^(37)^. Coexistently, a prospective study carried out in Brazil found that, in the perioperative period^(37)^, Lean Six Sigma is effective in improving the discharge process even in other contexts, such as Intensive Care Units (ICUs)^(46)^. Certainly, this is because Lean corroborates the re-evaluation of the operational performance of the entire system, thereby bringing about changes in terms of resources, technology and infrastructure^(41)^. This makes it possible to achieve an increase in the weekly elective surgical volume^(37)^, as the turnover time between operative procedures is reduced^(41, 42)^.

In the surgical context, the disorder of processes is seen as one of the factors responsible for causing a lack of beds in the postanesthetic recovery unit and, consequently, a delay in patient flow, culminating in prolonged discharge and use of hospital resources^(37)^. However, by optimizing this flow through the implementation of Lean Six Sigma, there is an increase in the number of hospital admissions due to the reduction in the patient’s recovery time after a surgical procedure^(43)^. This shows that surgical processes optimized in a continuous manner, i.e. achieving less processing time and variability, corroborate the stability and efficiency of systems for proposing safe and quality healthcare.

In short, it should be noted that by optimizing the flow of surgical patients through Lean and Six Sigma, it is possible to reduce the waiting time and turnover of surgeries, increase the volume of surgical admissions and ensure efficient processing and use of resources and systems^(41, 42, 43)^, with an influence on reducing the length of stay of the patient in the healthcare organization.

### Work process

The work process is one of the indicators of success in implementing Lean and/or Six Sigma^(39, 44)^. Optimized work processes, i.e. with less processing time and variability, reduce the time it takes to start implementing the treatment plan after surgery^(44)^ and a reduction in the rate of delayed surgeries due to lack of materials^(39)^. This means that Lean and/or Six Sigma not only have the potential to maximize the achievement of health outcomes that are favorable to the patient, but also favorable to healthcare professionals and organizations.

However, it is not enough for favorable health outcomes to be achieved without ensuring patient safety, since this indicator is directly related to the quality of health care^(47)^. It is therefore imperative that the optimization of perioperative processes becomes a reality, since organized work processes denote indicators of waste and inefficiency and lead to the establishment of systems that add value, reducing costs for the healthcare organization and improving the quality and effectiveness of the system^(39, 44)^.

In line with these findings, studies that evaluated the relationship between Lean and the optimization of hospitalization processes^(6)^ and patient discharge^(6, 48)^ showed that Lean also makes it possible to optimize the work process. In addition, this methodology has the potential to help establish a collaborative interprofessional work process in which the autonomy of professionals in patient care is observed, as well as shared decision-making and improved communication^(49)^.

However, given that Lean and/or Six Sigma corroborate the establishment of optimized processes, further studies should investigate the relationship between such optimization and a reduction in staff turnover, as well as its relationship with the flow of surgical patients.

### Length of stay

When concluding the influence of Lean and/or Six Sigma on the optimization of work processes and the flow of surgical patients, the repercussions of these aspects on the length of stay of patients should be noted^(38, 45)^.

In this sense, of the total number of studies included, three assessed the perioperative period and associated issues, such as patients’ perceptions of waiting times^(38)^ and the average number of days of hospitalization^(9)^. Lean and/or Six Sigma were found not only to improve patient perception of waiting times, but also to achieve a significant reduction in the average number of days spent in hospital^(9, 38)^. This highlights the fact that, to the extent that the methodologies help the patient achieve a positive health outcome, higher levels of patient satisfaction can also be seen.

When evaluating the influence of Lean on the establishment of safe processes and the achievement of better results, a cohort study conducted in the United Kingdom showed that the length of a patient’s stay in hospital is associated with a significant risk of patient safety incidents. This means that the longer the length of stay, the greater the risk of adverse events^(45)^. For this reason, by optimizing work processes and the flow of surgical patients with Lean, there is a contribution to reducing length of stay, denoting indicators of reliability and efficiency.

These indicators also help to ensure that patients are better informed about their treatment plan^(38)^, so that, by taking ownership of their treatment in detail and with information, the chances of promoting self-care in a more assertive manner are increased, which can contribute to reducing the hospital readmission.

Finally, although the amount of evidence is small, the studies emphasize the potential of Lean and/or Six Sigma to reduce the length of stay of surgical patients; a result in line with the findings of a study that evaluated Lean Six Sigma to reduce the length of stay of patients in the emergency department^(50)^. It is suggested that new studies be carried out that can attest to this relationship, especially by evaluating the perioperative period and the different periods that make it up. Indicators related to health care such as avoidability, the rate of surgical reoperations and rates of adverse events could be key to understanding this relationship.

### Limitations of the study

Even though this study was conducted with precision and methodological rigor, contingently, when contemplating the perioperative period, the synthesis of evidence may not have considered the specificities of each operative period. Nevertheless, this study achieved its objective and contributed significant findings.

### Contributions to healthcare

This study showed that Lean and/or Six Sigma contribute to optimizing processes in the perioperative period. This has a number of repercussions in the organizational, managerial and clinical spheres. In the organizational sphere, these methodologies corroborate governability by guaranteeing the sustainability of systems. In the managerial sphere, they contribute to the implementation of quality management tools and even to the incorporation of a culture of continuous improvement into the organizational culture. Finally, in the clinical sphere, they ensure that care can be provided in a qualified manner to achieve a satisfactory patient experience.

## FINAL CONSIDERATIONS

Most of the studies concluded that Lean and/or Six Sigma make a significant contribution to optimizing perioperative processes, reducing time and process variability. In addition, they organize the work process, optimize the flow of surgical patients and reduce length of stay, guaranteeing better patient experiences and favorable health outcomes.

In addition, they make it possible to identify potential problems to recognize them and propose solutions that can make patient-centered care possible. Lean and/or Six Sigma guarantee the provision of lean patient care, operated with the necessary resources and at the optimum time, in order to avoid unnecessary human movement and transport, maximizing the achievement of favorable health outcomes for the patient, the professionals and the healthcare organization. In addition, they allow the patient to take ownership of their treatment plan with a greater level of information, thereby increasing the chances of promoting self-care in a more assertive manner, with a possible influence on reducing the chances of hospital readmission.
