# Supplementary material for: Lean and/or Six Sigma for process optimization in the perioperative period: an integrative review
Source: Rev Bras Enferm. 2024 Jun 14;77(2):e20230431. doi: 10.1590/0034-7167-2023-0431 (PMC11178310; doi:10.1590/0034-7167-2023-0431)
Supplement: Translation

# Translation: Lean e/ou Six Sigma para otimização de processos no período perioperatório: revisão integrativa

**DOI:** 10.1590/0034-7167-2023-0431pt

**Authors:** Lucas Gardim, Fernanda Rodrigues dos Santos, Bruna Moreno Dias, Lívia Barrionuevo El Hetti Fuentes, Renata Cristina de Campos Pereira Silveira, Andrea Bernardes

**Affiliations:** I Universidade de São Paulo. Ribeirão Preto, São Paulo, Brasil

**Keywords:** Centros Cirúrgicos, Gestão da Qualidade Total, Otimização de Processos, Período Perioperatório, Saúde

## Abstract

**Objetivo:**

Analisar as evidências acerca da influência do Lean e/ou Six Sigma para otimização de processos no período perioperatório.

**Métodos:**

Revisão Integrativa realizada nas bases de dados MEDLINE (PubMed), Web of Science, EMBASE, CINAHL, Scopus e LILACS, a respeito do uso do Lean e/ou Six Sigma para otimização de processos no perioperatório. Os estudos incluídos foram analisados em três categorias temáticas: fluxo de pacientes cirúrgicos, processo de trabalho e tempo de permanência.

**Resultados:**

A amostra final foi composta por dez estudos, os quais contemplaram todos os períodos operatórios. Lean e/ou Six Sigma contribuem de forma expressiva para a otimização dos processos no perioperatório.

**Considerações finais:**

Lean e/ou Six Sigma otimizam processos perioperatórios em vista da maximização do alcance de indicadores de estabilidade dos sistemas, tornando possível a identificação de potenciais problemas para o reconhecimento e proposição de soluções que possam viabilizar a instituição de um cuidado centrado no paciente.

## INTRODUÇÃO

Evidências científicas demonstram a efetividade do Lean e/ou Six Sigma quando aplicados ao cenário da saúde^(1, 2)^. No contexto perioperatório, os processos de trabalho e de pacientes possuem grande complexidade^(3)^, haja vista abrangência de diversos profissionais e interlocução com diferentes setores intra-hospitalares. Por essa razão, faz-se imperiosa a otimização de processos relativos ao fluxo de trabalho e de pacientes, sobretudo com o objetivo de reduzir o tempo de permanência e alcançar resultados positivos relacionados à saúde.

Em meados da década de 80, com origem no Sistema Toyota de Produção, o Lean foi estabelecido como uma filosofia que tem como foco a redução de desperdícios (tempo de espera, movimento humano desnecessário, transporte desnecessário, retrabalho, ultraprocessamento, superprodução e estoque) e a instituição de processos que agregam valor^(4)^. Em outra perspectiva, originado na Motorola, Six Sigma é uma metodologia que tem como foco a redução da variabilidade nos processos. Dada a complementaridade, a filosofia Lean e a metodologia Six Sigma foram congregadas em “Lean Six Sigma”^(5)^.

Quando aplicadas a processos de otimização, alta de paciente, unidades de terapia intensiva, departamento de emergência, sala de cirurgia e demais unidades, Lean e/ou Six Sigma viabilizam a redução do tempo de permanência^(6)^, contribuindo para o aprimoramento da segurança do paciente, minimização do desconforto percebido e até mesmo redução dos custos para a organização de saúde^(7, 8, 9, 10, 11, 12, 13, 14)^.

Para além disso, tratando-se do perioperatório, estudos indicaram a influência da implementação do Lean e/ou Six Sigma na redução do tempo de rotação da sala de cirurgia, garantindo a prestação de um serviço eficiente^(15, 16, 17)^. Com isso, para proposição de um gerenciamento efetivo, fica evidente a imprescindibilidade da identificação de limitações processuais que possam viabilizar a otimização dos processos, isto é, a redução do tempo e da variabilidade processual, aprimorando a eficiência da sala de cirurgia^(18, 19)^.

Ainda que a assistência cirúrgica consuma uma grande quantitativo de recursos tecnológicos e onere vigorosamente a organização de saúde^(20)^, nem sempre todas as despesas sucedidas para realização dos procedimentos cirúrgicos são de fato necessárias, tendo em vista que se relacionam diretamente ao modo em que as salas de cirurgia são gerenciadas. Todavia, a adoção do Lean e/ou Six Sigma corrobora potencialmente o alcance da sistematização dos processos e a redução dos custos para a organização hospitalar, não apenas transformando a qualidade do atendimento, mas também aumentando os índices de satisfação de pacientes e de profissionais^(19, 21, 22, 23)^.

Nesse sentido, tendo em vista o potencial do Lean e/ou Six Sigma para o alcance de excelentes indicadores de qualidade no perioperatório^(24, 25)^, faz-se imprescindível a síntese das evidências científicas que comprovem tal efetividade, a fim de endossar a implementação de tais metodologias em diferentes organizações hospitalares, especialmente no que diz respeito à assistência cirúrgica, com influência sobre os desfechos de saúde alcançados pelos pacientes.

## OBJETIVO

Analisar as evidências acerca da influência do Lean e/ou Six Sigma para otimização de processos no período perioperatório.

## MÉTODOS

Revisão integrativa fundamentada no referencial metodológico de Whittemore e Knafl (2005)^(26)^ e apresentada conforme *Preferred Reporting Items for Systematic Review and Meta-Analysis* (PRISMA)^(27)^.

A questão norteadora foi elaborada por intermédio do acrônimo PICo (P= População; I= Interesse; Co= Contexto)^(28)^. Desse modo, atribuiu-se “P” à otimização de processos; “I” ao Lean e/ou Six Sigma; e “Co” ao período perioperatório, compreendendo pré, intra e pós-operatório. Assim, estabeleceu-se a seguinte questão norteadora: “Qual a influência do Lean e/ou Six Sigma para otimização de processos no período perioperatório?”.

A busca foi conduzida durante o mês de agosto de 2021 e atualizada em abril de 2023 nas bases de dados: *Medical Literature Analysis and Retrieval System Online* (MEDLINE/PubMed), Web of Science, EMBASE, *Cumulative Index to Nursing and Allied Health Literature* (CINAHL), Scopus e Literatura Latino-Americana e do Caribe em Ciências da Saúde (LILACS).

Para isso, foram utilizados descritores controlados e não controlados em conformidade com o *Medical Subject Headings* (MeSH), Emtree, Títulos de Assuntos do CINAHL e Descritores em Ciências da Saúde (DeCS). A fim de garantir a sensibilidade da estratégia de busca nas bases de dados selecionadas, tal processo foi realizado com o apoio de uma bibliotecária com *expertise* em busca bibliográfica. O Quadro 1 apresenta as estratégias de busca aplicadas em cada base de dados.

**Quadro 1:** Estratégia de busca nas bases de dados selecionadas

Base de dados	Estratégia de Busca
MEDLINE (PubMed)	(“Total Quality Management” OR “Lean Healthcare” OR “Lean System” OR “Lean methodology” OR “Lean Six Sigma” OR “Six Sigmas” OR “Six Sigma”) AND (Surgicenter* OR “Surgery Department”)
*Web of Science*	(“Total Quality Management” OR “Lean Healthcare” OR “Lean System” OR “Lean methodology” OR “Lean Six Sigma” OR “Six Sigmas” OR “Six Sigma”) AND (Surgicenter* OR “Surgery Department”)
EMBASE	(“Total Quality Management” OR “Lean Healthcare” OR “Lean System” OR “Lean methodology” OR “Lean Six Sigma” OR “Six Sigmas” OR “Six Sigma”) AND (Surgicenter* OR “Surgery Department”)
CINAHL	(“Total Quality Management” OR “Lean Healthcare” OR “Lean System” OR “Lean methodology” OR “Lean Six Sigma” OR “Six Sigmas” OR “Six Sigma”) AND (Surgicenter* OR “Surgery Department”)
SCOPUS	(“Total Quality Management” OR “Lean Healthcare” OR “Lean System” OR “Lean methodology” OR “Lean Six Sigma” OR “Six Sigmas” OR “Six Sigma”) AND (Surgicenter* OR “Surgery Department”)
LILACS	(“Total Quality Management” OR “Lean Healthcare” OR “Lean System” OR “Lean methodology” OR “Lean Six Sigma” OR “Six Sigmas” OR “Six Sigma”) AND (Surgicenter* OR “Surgery Department”)

No que tange à seleção de manuscritos, foram considerados como critérios de inclusão: artigos originais, publicados de 2010 a 2023 e sem recorte para o idioma, que avaliaram a influência do Lean e/ou Six Sigma para otimização de processos no período perioperatório. Foram excluídas revisões de literatura e literatura cinzenta.

A decisão pelo recorte temporal se deu em virtude da linha do tempo de implementação do Lean e/ou Six Sigma no segmento da saúde. De fato, não há consenso na literatura a respeito do ano exato em que o processo de implementação foi iniciado; todavia, estima-se que se deu por volta dos anos 2000. A exemplo dos Estados Unidos, o primeiro evento para difusão do Lean e/ou Six Sigma ocorreu em 2010^(29)^, ao passo que, no Brasil, estima-se que o processo seu deu a partir de 2013^(30)^, denotando desigualdade não apenas na implementação, mas, ainda, na difusão do tema. Por essa razão, tratando-se de um processo de gestão da mudança, no que diz respeito à cultura das organizações de saúde para implementação do Lean e/ou Six Sigma, estimou-se que os indicadores relativos à implementação puderam ser denotados em torno de 2010, especialmente se tratando do perioperatório.

Os estudos identificados nas bases de dados foram exportados para o *Rayyan QCRI* ^(31)^ para remoção de estudos duplicados e triagem. A partir disso, três etapas de seleção foram sucedidas por dois revisores de forma independente.

Na primeira etapa, após a remoção dos duplicados, foram avaliados os títulos e resumos dos estudos identificados. Em seguida, realizou-se a leitura na íntegra para confirmar a sua elegibilidade. Evidencia-se que as divergências entre os revisores foram solucionadas por meio de reuniões entre os pares. Nas ocasiões em que as divergências não foram solucionadas, um terceiro revisor com expertise no tema foi convocado. Ao final, realizou-se a extração de dados dos estudos selecionados para amostra de forma independente, considerando: autoria, ano de publicação e país em que o estudo foi conduzido; metodologia utilizada (Lean Healthcare, Six Sigma ou Lean Six Sigma) e período operatório (pré-operatório, intraoperatório e/ou pós-operatório); ferramentas utilizadas e população; resultados alcançados e desfecho.

Para a amostra final, analisou-se a força de evidência, conforme proposto por Melnyk & Fineout-Overholt (2011)^(32)^. Nesse sentido, os estudos foram avaliados de acordo com três hierarquias, a saber: (1) Intervenção ou Diagnóstico / Teste Diagnóstico, com sete níveis; (2) Prognóstico / Predição ou Etiologia, com cinco níveis; e (3) Significado, com cinco níveis. Para as três hierarquias, quanto menor o nível, mais forte a evidência^(32)^.

Ademais, a fim de avaliar a qualidade metodológica, utilizou-se o *Critical Appraisal Checklist for Studies Reporting Prevalence Data*, recomendado pelo JBI^(33)^. Assim sendo, para os estudos transversais, foi utilizada a ferramenta *JBI Critical Appraisal Checklist for Analytical Cross Sectional Studies* ^(34)^; para estudos de coorte, empregou-se a ferramenta *JBI Critical Appraisal Checklist for Cohort Studies* ^(34)^; para estudos qualitativos, foi utilizada a ferramenta *JBI Critical Appraisal Checklist for Qualitative Research* ^(35)^; para os estudos quase experimentais, foi aplicada a ferramenta *JBI Critical Appraisal Checklist for Quasi-Experimental Appraisal Tool* ^(36)^. Evidencia-se que, na avaliação metodológica dos estudos, é notório que um maior quantitativo de respostas classificadas como “Sim” indica melhor qualidade metodológica, segundo ferramentas propostas pelo JBI^(33)^.

Ressalta-se que os processos de avaliação da força de evidência e da qualidade metodológica foram realizados por dois revisores de maneira independente. As discordâncias dos resultados preliminares foram solucionadas por um terceiro revisor com expertise metodológica. Em seguida, os dados foram analisados a partir de análise descritiva e de conteúdo, que incluiu contagens de frequência e descrições de fluxo de pacientes cirúrgicos, processo de trabalho e tempo de permanência.

## RESULTADOS

Foram identificados 1889 registros nas seis bases de dados. Seguindo o fluxo de seleção de artigos, dez artigos foram incluídos nesta revisão^(9, 37, 38, 39, 40, 41, 42, 43, 44, 45)^, de acordo com a Figura 1.

**Figura 1:** Fluxograma de seleção dos estudos segundo fluxograma PRISMA^(27)^

Dentre os estudos incluídos, quatro (40%) foram desenvolvidos do continente Americano (um no Brasil^(39)^, um no Canadá^(43)^, um nos Estados Unidos^(37)^ e um no Peru^(40)^), quatro (40%) foram desenvolvidos no continente Europeu (um na Irlanda^(38)^, um na Itália^(9)^, um no Reino Unido^(45)^ e um na Suíça^(41)^) e dois (20%) foram realizados no continente Asiático (um na Arábia Saudita^(44)^ e um em Taiwan^(42)^). No que tange aos anos de publicação, notou-se o predomínio de estudos publicados no ano de 2015 (n=2), correspondendo a 20% da amostra.

Da amostra final, ressalta-se que cinco estudos (50%) adotaram Lean Healthcare^(38, 39, 41, 43, 45)^ para a avaliar a otimização de processos, três (30%) aplicaram Six Sigma^(37, 42, 44)^ e dois (20%) Lean Six Sigma^(9, 40)^. A partir disso, observou-se que as referidas metodologias possuem diversidade de aplicação nos períodos operatórios, haja vista que todos foram contemplados, a saber: pré-operatório (n=5)^(9, 37, 38, 39, 40)^, intraoperatório (n=6)^(9, 37, 38, 40, 41, 42)^ e pós-operatório (n=6)^(9, 37, 38, 43, 44, 45)^. Enfatiza-se que a soma dos estudos ultrapassa o número de incluídos na amostra final, considerando que três estudos contemplam o período perioperatório^(9, 37, 38)^ e um engloba os períodos pré e intraoperatório^(40)^. O Quadro 2 apresenta a síntese dos manuscritos analisados.

**Quadro 2:** Síntese dos artigos incluídos na revisão integrativa da literatura

Período Operatório	Autoria / País	Metodologia	Intervenção	Resultados	Desfecho: Otimizou os Processos?	Nível de Evidência
**Perioperatório**	Amato-Vealey et al., 2012^(37)^ Estados Unidos	Six Sigma	Ferramentas: mapeamento do fluxo do paciente, *Brainstorming*, visita multidisciplinar. População: não especificado.	A implementação do Six Sigma resultou em melhoria no processo de alta e garantiu a capacidade de manter um fluxo contínuo de pacientes cirúrgicos sem incorrer em custos financeiros.	Sim	VI*
	Montella et al., 2017^(9)^ Itália	Lean Six Sigma	Ferramentas: *Define-Measure-Analyze-Improve-Control* (*DMAIC*), mapeamento de processos, *brainstorming* e espinha de peixe. População: 20.000 pacientes.	As ações resultaram em uma redução significativa do número médio de dias de internação (de 45 para 36 dias). Houve, ainda, a redução da porcentagem de pacientes colonizados por bactérias sentinelas (de 0,37% para 0,21%).	Sim	II*
	Ullah et al., 2020^(38)^ Irlanda	Lean Healthcare	Ferramentas: 5s, Kaizen, sistema puxado e empurrado e mapeamento de processos. População: Fase 1: 100 pacientes e 20 funcionários (*clerical*, administrativo e equipe de saúde) entrevistados para a compreensão de suas perspectivas nas áreas que exigiam atenção para melhorar a prestação de serviços e a eficiência; Fase 2: 100 pacientes.	Os resultados demonstram a “percepção” do paciente sobre o tempo de espera. Na fase 2, a percepção melhorou com 83% dos pacientes em comparação aos 67% da fase 1; a percepção de tempo de espera para revisão médica também melhorou significativamente, com 26% de pacientes revisados na fase 2 em comparação à 0% na fase 1. Apenas 7% de pacientes tiveram uma permanência total de 6-8h na fase 2 em comparação aos 33% na fase 1. Houve satisfação em diferentes domínios, percepções de privacidade e o tratamento geral também foi aprimorado.	Não se pode afirmar que houve otimização dos processos.	VI*
**Pré-operatório**	Godinho Filho et al., 2015^(39)^ Brasil	Lean Healthcare	Ferramentas: 5s e *Single Minute Exchange of Dies* (*SMED*). População: gestor de qualidade (n=1), analista de qualidade (n=1), coordenador do Centro de Materiais e Esterilização (CME) (n=1), enfermeiro chefe do centro cirúrgico (n=1), consultores externos (não especificado), gerente de solução (n=1), consultores analistas (n=2).	Uma redução significativa de 94% no índice de atraso cirúrgico devido à falta de materiais do Centro de Esterilização de Materiais (CME) foram observados após o evento Kaizen. Finalmente, uma redução significativa na infecção pós-operatória pôde ser observada devido à melhora do fluxo e procedimentos operacionais padronizados, saindo de 1 a 1,5% para 0,21%.	Sim	VI*
**Pré-operatório e Intraoperatório**	Iverson et al., 2021^(40)^ Peru	Lean Six Sigma	Ferramentas: *Plan-Do-Study-Act* (*PDSA*), mapeamento de processos e espinha de peixe. População: 1- Pré-intervenção: 75 pacientes (53 emergência e 22 eletivos). 2- Pós-intervenção: 109 pacientes (111 emergência e 31 eletivos).	Os pacientes (87%) na nova lista de espera tinham todos os dados clínicos relevantes documentados, uma melhoria de 13,3% para a lista pré-existente. O tempo da admissão à alta para todas as cirurgias melhorou de 5 para 4 dias (p<0,05) após a intervenção.	Sim	II*
**Intraoperatório**	Amati et al., 2022^(41)^ Suíça	Lean Healthcare	Ferramentas: Gemba *walks*, mapeamento de processos, análise de causa raiz e *Single Minute Exchange of Dies* (*SMED*). População: equipe multidisciplinar (um *chair of anesthesia*, um enfermeiro anestesista, um técnico cirúrgico, um *housekeeper*, um secretário administrativo da sala de cirurgia, dois cirurgiões e dois enfermeiros-chefes, representando as especialidades de ginecologia e cirurgia geral).	O tempo de troca entre as operações foi reduzido em 17 min para ginecologia e 15 min para cirurgia geral (25% em média), sem alterações em termos de infraestrutura, tecnologia ou recursos.	Sim	VI*
	Leu et al., 2013^(42)^ Taiwan	Six Sigma	Ferramentas: mapeamento de processos. População: Fase 1: 1246 pacientes. Fase 2: 1266 pacientes.	A redução do tempo de espera para cada grupo de estudo foi de 0,54, 4,23 e 7,82 minutos, respectivamente. O tempo médio de rotatividade da cirurgia também diminuiu significativamente para cada grupo (11,98, 16,4 e 19,72 minutos, respectivamente). O indicador de qualidade do Seis Sigma foi alterado de 3,35δ para 3,46δ, uma diferença de 0.11δ.	Sim	II*
**Pós-operatório**	Blouin-Delisle et al., 2018^(43)^ Canadá	Lean Healthcare	Ferramentas: *Define-Measure-Analyze-Improve-Control* (*DMAIC*), workshop Kaizen, mapeamento de processos. População: profissionais do *Project Care Unit*: enfermeiro chefe (n=2), enfermeiros (n=3), *clerical asisstant* (n=1), chefe de admissão (n=1), chefe de ala cirúrgica (n=1), agente responsável pela implementação dos conceitos Lean (n=1).	Hospital 1: O tempo de permanência na enfermaria de recuperação foi reduzido em 62 minutos (redução de 68%) e houve um aumento de cerca de 25% de todas as admissões feitas durante o dia após o projeto em comparação com o período anterior ao projeto. Hospital 2: Para o projeto HEJ Lean, o tempo na enfermaria de recuperação foi reduzido em 6 minutos (redução de 29%).	Sim	VI*
	Bouras, 2015^(44)^ Arábia Saudita	Six Sigma	Ferramentas: mapeamento de processos, espinha de peixe, análise de causa raiz, diagrama de causa e efeito, *brainstorming*. População: não especificado.	Os resultados evidenciam a redução do tempo de ciclo, impactando na redução do tempo necessário para contatar os médicos; na minimização do tempo necessário para a realização de casos urgentes e para o processo de encaminhamento do paciente; além da redução do tempo de atraso antes de iniciar a execução do plano de tratamento.	Sim	IV**^†^**
	McCulloch et al., 2010^(45)^ Reino Unido	Lean Healthcare	Ferramentas: 5S, mapeamento de processos, gestão visual e *Plan-Do-Check-Action* (*PDCA*). População: 1- Pré-intervenção: 607 pacientes. 2- Pós-intervenção: 602 pacientes.	Enfoque no estabelecimento de processos seguros e melhores resultados. A proporção de pacientes que necessitam de transferência para outras enfermarias caiu de 27% para 20%. O tempo de permanência se mostrou como fator de risco mais importante para incidentes de segurança do paciente.	Não se pode afirmar que houve otimização dos processos.	IV*

*Intervenção ou Diagnóstico / Teste Diagnóstico; †Prognóstico / Predição ou Etiologia.

No que compreende à análise de qualidade, dos estudos transversais (n=4), dois receberam um total de seis respostas “sim”; dos estudos qualitativos (n=2), apenas um recebeu o total de oito respostas “sim”; dos estudos com delineamento quase experimental (n=3), dois receberam o total de oito respostas “sim”; e, por fim, do estudo coorte (n=1), o artigo incluído recebeu um total de nove respostas “sim”. O Quadro 3 evidencia a avaliação metodológica discriminada por ferramenta e delineamento metodológico. Ressalta-se que nenhum dos estudos incluídos recebeu o total de respostas “sim”, considerando a quantidade de perguntas compreendidas em cada instrumento.

**Quadro 3:** Avaliação Metodológica por meio do JBI Critical Appraisal Checklist for Studies Reporting Prevalence Data

**Transversa** ^l(34)^	**Q1**	**Q2**	**Q3**	**Q4**	**Q5**	**Q6**	**Q7**	**Q8**	**Q9**	**Q10**	**Q11**	**Total (Sim)**
Amato-Vealey et al., 2012^(37)^	N*	S^†^	S	S	N	I^‡^	S	I	-	-	-	4
Blouin-Delisle et al., 2018^(43)^	S	S	S	S	I	I	S	S	-	-	-	6
Bouras, 2015^(44)^	N	I	S	S	N	N	S	S	-	-	-	4
Ullah et al., 2020^(38)^	S	S	S	S	N	N	S	S	-	-	-	6
**Qualitativo** ^(36)^												
Amati et al., 2022^(41)^	S	S	S	S	S	S	I	NA^§^	S	S	-	8
Godinho Filho et al., 2015^(39)^	S	S	S	S	S	S	I	I	N	S	-	7
**Quase Experimental** ^(36)^												
Iverson et al., 2021^(40)^	S	S	S	N	S	S	S	S	S	-	-	8
Leu et al., 2013^(42)^	S	S	S	N	S	S	S	S	S	-	-	8
Montella et al., 2017^(9)^	S	S	S	N	N	S	S	S	S	-	-	7
**Coorte** ^(33)^												
McCulloch et al., 2010^(45)^	S	S	S	I	S	S	S	S	S	NA	S	9

*N= Não; ^†^S= Sim; ^‡^I= Incerto; ^§^NA= Não Aplicável.

## DISCUSSÃO

Para a análise das publicações incluídas neste estudo e o delineamento do constructo teórico, os artigos selecionados foram organizados em três categorias temáticas, a saber: fluxo de pacientes cirúrgicos^(37, 40, 41, 42, 43)^, processo de trabalho^(39, 44)^ e tempo de permanência^(9, 38, 45)^.

### Fluxo de Pacientes Cirúrgicos

Do total de estudos incluídos nesta revisão, cinco analisaram a influência do Lean e/ou Six Sigma no que diz respeito à otimização do fluxo de pacientes cirúrgicos^(37, 40, 41, 42, 43)^.

Tratando-se do contexto perioperatório, faz-se imprescindível que o fluxo de pacientes cirúrgicos seja otimizado para que o tempo de espera em procedimentos cirúrgicos seja minimizado, até mesmo para os gerais eletivos, tendo em vista a experiência e a segurança do paciente. Nesse sentido, considerando os princípios da cultura de melhoria contínua abarcados pelo Lean, deve-se sempre almejar a promoção de um cuidado enxuto ao paciente, garantindo os recursos necessários e no tempo ideal, evitando movimento humano e transporte desnecessários.

De fato, o Six Sigma tem o potencial de garantir um fluxo contínuo de pacientes cirúrgicos, reduzindo o tempo de admissão à alta para diversos procedimentos cirúrgicos^(37)^. Coexistentemente, estudo prospectivo realizado no Brasil avaliou que, assim como no perioperatório^(37)^, Lean Six Sigma é eficaz para aprimorar o processo de alta até mesmo em outros contextos, como em Unidades de Terapia Intensiva (UTI)^(46)^. Certamente, isso ocorre tendo em vista que o Lean corrobora a reavaliação do desempenho operacional de todo o sistema, ocasionando, com isso, mudanças em termos de recursos, tecnologia e infraestrutura^(41)^. Assim, torna-se possível alcançar um aumento do volume cirúrgico eletivo semanal^(37)^, na medida que o tempo de rotatividade entre os procedimentos operatórios são reduzidos^(41, 42)^.

No contexto cirúrgico, a desordem dos processos é tida como um dos fatores responsáveis por ocasionar a falta de leitos na unidade de recuperação pós-anestésica e, consequentemente, o atraso no fluxo dos pacientes, culminando no prolongamento da alta e do uso dos recursos hospitalares^(37)^. Todavia, ao otimizar tal fluxo por meio da implementação do Lean Six Sigma, nota-se o aumento do número de admissões hospitalares em virtude da redução do tempo do paciente na recuperação após procedimento cirúrgico^(43)^. Isso demonstra que processos cirúrgicos otimizados de modo contínuo, isto é, alcançando menor tempo e variabilidade de processamento, corroboram a estabilidade e eficiência dos sistemas para a proposição de uma assistência em saúde segura e de qualidade.

Em suma, destaca-se que, com a otimização do fluxo de pacientes cirúrgicos alcançada através do Lean e Six Sigma, é possível diminuir o tempo de espera e de rotatividade das cirurgias, aumentar o volume de admissões operatórias e garantir a eficiência do processamento e da utilização de recursos e sistemas^(41, 42, 43)^, com influência sobre a redução do tempo de permanência do paciente na organização de saúde.

### Processo de Trabalho

Tem-se o processo de trabalho como um dos indicadores de sucesso da implementação do Lean e/ou Six Sigma^(39, 44)^. Processos de trabalho otimizados, isto é, com menor tempo e variabilidade de processamento, propiciam a redução do tempo para o início da execução do plano de tratamento após a cirurgia^(44)^ e a redução na taxa de atraso nas cirurgias devido à falta de materiais^(39)^. Isso significa que Lean e/ou Six Sigma apresentam não somente potencial para maximizar o alcance de resultados de saúde favoráveis ao paciente, mas ainda são favoráveis aos profissionais e às organizações de saúde.

Todavia, não é suficiente que desfechos favoráveis de saúde sejam alcançados sem a garantia de segurança para o paciente, visto que tal indicador se relaciona diretamente à qualidade dos cuidados em saúde^(47)^. Por isso, é premente que a otimização de processos perioperatórios se torne uma realidade, já que processos de trabalho organizados denotam indicadores de desperdício e de ineficiência menores e levam à instauração de sistemas que agregam valor, reduzindo os custos para a organização de saúde e aprimorando a qualidade e a eficácia do sistema^(39, 44)^.

Em encontro aos achados, os estudos que avaliaram a relação do Lean com a otimização dos processos de hospitalização^(6)^ e alta dos pacientes^(^6,48^)^ evidenciaram que o Lean também possibilita a otimização do trabalho. Além disso, essa metodologia tem potencial para colaborar com a instauração de um processo de trabalho interprofissional colaborativo, em que se observa a autonomia de profissionais no acompanhamento do paciente, bem como a tomada de decisão compartilhada e o aprimoramento da comunicação^(49)^.

Não obstante, tendo em vista que Lean e/ou Six Sigma corroboram a instauração de processos otimizados, novos estudos devem investigar a relação entre tal otimização e a diminuição da rotatividade de profissionais, tanto quanto sua relação com o fluxo de pacientes cirúrgicos.

### Tempo de Permanência

Ao concluir a influência do Lean e/ou Six Sigma para a otimização de processos de trabalho e do fluxo de pacientes cirúrgicos, deve-se observar a repercussão de tais aspectos sobre o tempo de permanência dos pacientes^(38, 45)^.

Nesse sentido, do total de estudos incluídos, três avaliaram o período perioperatório e questões associadas, como, por exemplo, a percepção dos pacientes sobre o tempo de espera^(38)^ e o número médio de dias de internação^(9)^. Constatou-se que Lean e/ou Six Sigma não só melhoram a percepção do paciente no que diz respeito ao tempo de espera, mas, ainda, o alcance de uma redução significativa do número médio de dias de internação^(9, 38)^. Isso destaca que, na medida em que as metodologias propiciam o alcance do desfecho positivo de saúde por parte do paciente, maiores índices relativos à sua satisfação também podem ser notados.

Ao avaliar a influência do Lean para o estabelecimento de processos seguros e para o alcance de melhores resultados, o estudo coorte conduzido no Reino Unido evidenciou que o tempo de permanência do paciente no hospital está associado a um risco significativo para o alcance de incidentes relativos à sua segurança. Isso significa que, quanto maior o tempo de permanência, maior o risco de eventos adversos^(45)^. Por essa razão, ao otimizar processos de trabalho e do fluxo de pacientes cirúrgicos com Lean, é evidente a existência de uma contribuição para a redução do tempo de permanência, denotando indicadores de confiabilidade e eficiência.

Não obstante, tais indicadores contribuem para que os pacientes estejam mais bem informados sobre seu plano de tratamento^(38)^, de modo que, ao se apropriar sobre os procedimentos de seu tratamento com o detalhamento de informações, aumentam-se as chances da promoção do autocuidado de maneira mais assertiva, podendo contribuir para a diminuição da reinternação hospitalar. Por fim, embora o quantitativo de evidências seja reduzido, os estudos ressaltam o potencial do Lean e/ou Six Sigma para diminuição do tempo de permanência dos pacientes cirúrgicos; resultado em consonância com os achados de estudo que avaliou Lean Six Sigma para redução do tempo de permanência dos pacientes no departamento de emergência^(50)^. Sugere-se o desenvolvimento de novos estudos que possam atestar tal relação, especialmente avaliando o perioperatório e, ainda, os diferentes períodos que o compõem. Indicadores relacionados aos cuidados em saúde como evitabilidade, índice de reabordagens cirúrgicas e índices de eventos adversos podem ser peças-chave para a compreensão de tal relação.

### Limitações do estudo

Ainda que este estudo tenha sido conduzido com precisão e rigor metodológico, contingentemente, ao contemplar o perioperatório, a síntese das evidências pode não ter considerado as especificidades de cada período operatório. Apesar disso, este estudo alcançou seu objetivo e contribuiu com descobertas significativas.

### Contribuições para a área da Saúde

Este estudo evidenciou que Lean e/ou Six Sigma contribuem para a otimização de processos no perioperatório. Com isso, diversas repercussões se sucedem no âmbito organizacional, gerencial e assistencial. No aspecto organizacional, tais metodologias corroboram a governabilidade por meio da garantia da sustentabilidade dos sistemas. No âmbito gerencial, contribuem para a implementação de ferramentas de gestão da qualidade e até mesmo para a incorporação da cultura de melhoria contínua à cultura organizacional. Por fim, no âmbito assistencial, asseveram a oferta do cuidado de modo qualificado para o alcance de uma experiência satisfatória pelo paciente.

## CONSIDERAÇÕES FINAIS

Majoritariamente, os estudos concluíram que o Lean e/ou Six Sigma contribuem de forma expressiva para a otimização de processos no perioperatório, reduzindo o tempo e a variabilidade dos processos. Além disso, organizam o trabalho, otimizam o fluxo de pacientes cirúrgicos e reduzem o tempo de permanência, garantindo o alcance de melhores experiências e resultados de saúde favoráveis ao paciente.

Adicionalmente, oportunizam a identificação de potenciais problemas para o reconhecimento e proposição de soluções que possam viabilizar a instituição de um cuidado centrado no paciente. Lean e/ou Six Sigma garantem a proposição de um cuidado enxuto ao paciente, operado com os recursos necessários e no tempo ideal, a fim de evitar movimento humano e transporte desnecessário, maximizando o alcance de resultados de saúde favoráveis ao paciente, aos profissionais e à organização de saúde. Além disso, permitem que o paciente se aproprie sobre seu plano de tratamento com maior nível de informações, aumentando, com isso, as chances da promoção do autocuidado de maneira mais assertiva com possível influência sobre a diminuição das chances de reinternação hospitalar.
